# Microscopy without a microscope? Opportunities and limitations of dental studies in anatomy during the COVID-19 pandemic in Germany

**DOI:** 10.12688/mep.20580.1

**Published:** 2025-01-09

**Authors:** Ann-Kathrin Peters, Karsten Winter, Elisabeth Witt, Hubert-Mario Kuntzsch, Sabine Löffler

**Affiliations:** 1 Institute of Anatomy, University of Leipzig, Leipzig, Saxony, Germany

**Keywords:** COVID-19 pandemic, regulations on the licensing of dentists, dental studies, virtual microscopy, evaluation

## Abstract

**Aim:**

During the COVID-19 pandemic, the microscopy course as part of the dental medical training in Leipzig, Germany, was offered both purely digitally and with reduced attendance time. The reformed regulations on the licensing of dentists (ZApprO; „Approbationsordnung für Zahnärzte und Zahnärztinnen“) now also provide the necessary legal basis for the curricular integration of digital teaching formats. Aim of this study is the detailed evaluation of the relevant courses and the resulting design of modern dental training.

**Methods:**

Evaluation is based on the subjective experiences of the students and the learning success objectively measured using a digital assessment course. Data was collected in the form of a trend study in two consecutive years after completion of the second semester.

**Results:**

Students were predominantly positive about the integrated approach, which combines classroom teaching with digital elements. The cohort that completed the course in this form also showed significantly better learning success than the purely digital cohort.

**Discussion:**

Regardless of the continued importance of practical training in dental studies, digital teaching formats developed as an emergency solution at the beginning of the pandemic should be evaluated with regard to their success and the existing potential should be further expanded in a targeted manner.

## Introduction

**Figure 1.**
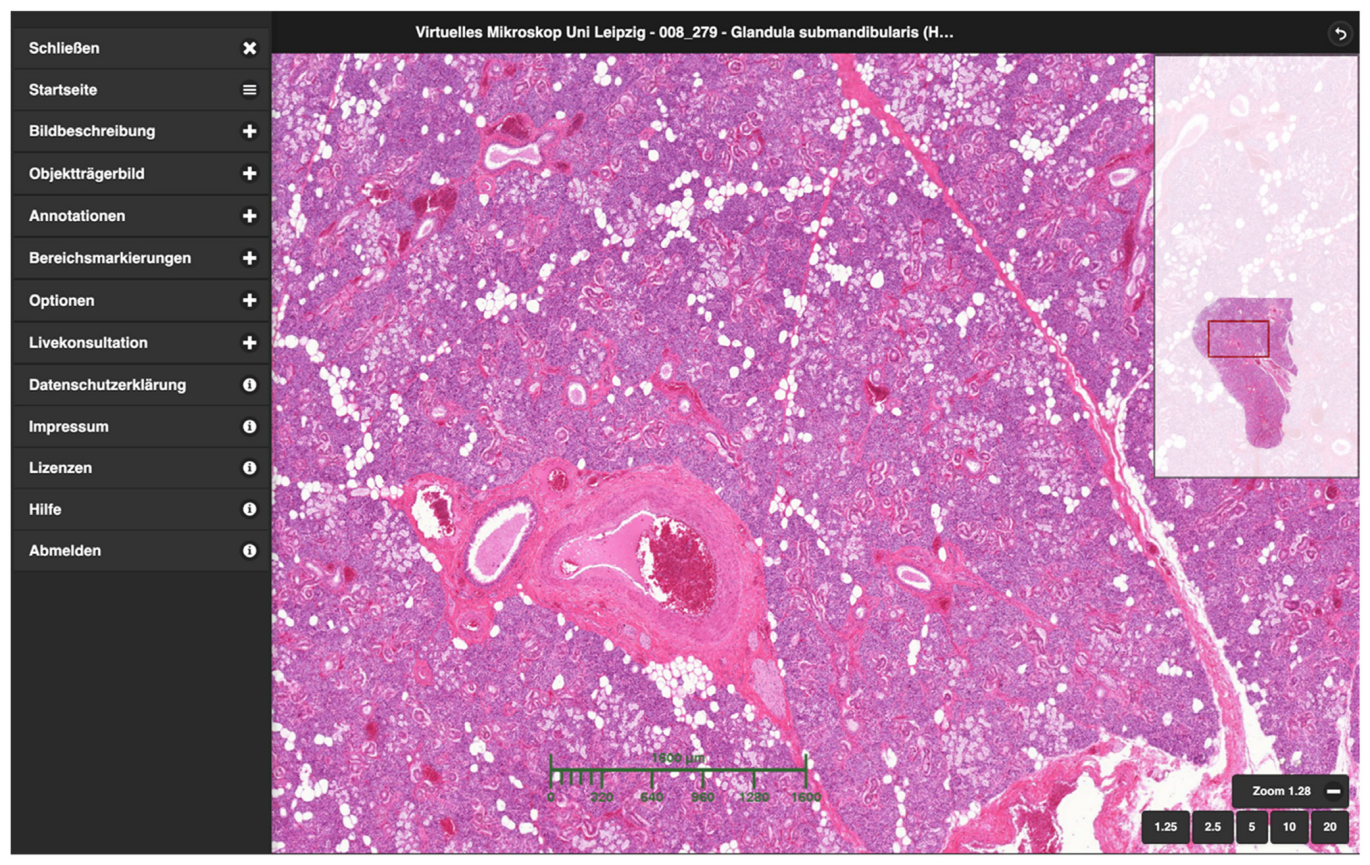
User interface of the CUVM using the example of the submandibular gland. On the left is the fold-out menu, on the right an overview image of the specimen and in the middle the freely navigable digital specimen viewer with a scale bar displayed.

Over the past 30 years, the teaching of microscopic and macroscopic anatomy has changed significantly ( Chapman *et al*., 2020; Trelease, 2016). Even before the outbreak of the COVID-19 pandemic, digital media were used intensively by students ( Friedrich *et al*., 2016). Technical developments in histology range from the first attempts to digitize analogue tissue preparations ( Silage & Gil, 1985) to virtual microscopy at the end of the 20th century ( Harris *et al*., 2001). In addition to traditional media, since 2017 all microscopic course specimens have been available to dental students on the central e-learning platform Core Unit Virtual Microscopy (CUVM, VMscope GmbH, Berlin, Germany) in digital form ( Figure 1). While some universities were already pursuing the integration of digital formats and traditional classroom teaching ( Cheng *et al*., 2017; Krippendorf & Lough, 2005; Mione *et al*., 2013), the concept in Leipzig, Germany, focused mainly on working with the light microscope. However, with the outbreak of the COVID-19 pandemic at the latest, the importance of digital teaching formats increasingly became the focus of interest.

**Digital teaching formats in the new ZApprO:** The reformed regulations on the licensing of dentists (ZApprO; “Approbationsordnung für Zahnärzte und Zahnärztinnen”), which came into force on October 1, 2020 and have been in force since the fall semester 2021, provided further impetus for a fundamental restructuring of dental studies in Germany. The reform pursued the goal of “prevention-oriented, interdisciplinary teaching” that works with “modern didactic methods” and “new training concepts” ( Kahl-Nieke & Vonneilich, 2018). The "Hochschulforum Digitalisierung“ (university forum digitalization) described the use of digital media as a core competence at an early stage and concluded that “this development of competence [...] cannot be expected as a by-product of subject-specific knowledge transfer”, but requires “targeted and systematic anchoring in curricula” ( Hochschulforum Digitalisierung, 2016). As long as there was an epidemic situation of national scope, practical courses could be supplemented and replaced by digital teaching formats (article 4, section 2, EpiZÄPrOAbwV; ordinance on deviation from the licensing regulations for dentists in the event of an epidemic situation of national importance; "Verordnung zur Abweichung von der Approbationsordnung für Zahnärzte bei einer epidemischen Lage von nationaler Tragweite“). With the last amendment to the new ZApprO of September 22, 2021 ("Bundesgesetzblatt“; BGBl, 2021, part I, number 67, page 4335), this legal formulation was also adopted for future teaching. However, practical exercises can now only be “accompanied by digital teaching formats” and can no longer be completely replaced (article 7, section 2, sentence 5, ZApprO).

**The microscopy course:** The study of histology in Leipzig extends over one academic year, divided into two course sections ( Studienordnung für den Studiengang Zahnmedizin an der Universität Leipzig, 2022). Starting with *general histology* in the first semester (part A), students learn the basics for studying *specific organ systems* in the second semester (part B). The theoretical knowledge is taught in a lecture in the first semester. For the practical courses, pure classroom teaching with light microscopes is planned. Both course sections are completed with a 45-minute multiple choice exam. In terms of content, the learning objectives are based on the national competence-based catalog of learning objectives in dentistry (NKLZ), which defines a “core curriculum” for the subject based on the current regulations on the licensing of dentists ( Nationaler Kompetenzbasierter Lernzielkatalog Zahnmedizin, 2023).

Since the second part of the course had to be conducted purely digitally at the beginning of the pandemic and the relevant course content later also corresponds to the examination content of the preliminary dental examination, the results presented below focus on the second semester.

At many universities, studying using a light microscope is still the standard. However, as digitalization has progressed, work with virtual tissue specimen has also developed. These can either be integrated into face-to-face teaching ( Mione *et al*., 2013) or used as part of a pure *e-learning* ( Darici *et al*., 2021) or *blended learning* approach ( Cheng *et al*., 2017). The latter is a combination of face-to-face and computer-based instruction ( Graham, 2006). During the pandemic, the microscopic course in Leipzig moved along a continuum between these two extremes: an exclusively e-learning phase (spring semester 2020) was followed by a phase with reduced attendance time and supplementary digital offerings (fall semester 2020 and 2021). In the spring semester 2022, there was a complete return to pure face-to-face teaching in its original form.

Even if there is talk of an accelerated digitalization of study programs in connection with the pandemic ( Hertling *et al*., 2022), discrepancies between students' wishes regarding digital offerings and the prevailing curricula have already been observed ( Stoehr *et al*., 2021). Does a return to the original structures therefore stand in contrast to recent experiences and developments?

In addition, the question arises how to the design modern teaching, as required by the reformed ZApprO ( Kahl-Nieke & Vonneilich, 2018). The multi-perspective evaluation of the microscopy course during the COVID-19 pandemic presented here should make it possible to answer these questions. Both the subjective experiences of the students (online evaluation form) and their objective learning success (digital identification course of easily confusable specimens) are analyzed and evaluated.

## Methods

**Evaluation form:** As part of the evaluation of the purely digital spring semester 2020 and the partially face-to-face spring semester 2021, dental students enrolled in the microscopic anatomy courses in the second semester were surveyed. The two consecutive years A (start of studies in fall semester 2019) and B (start of studies in fall semester 2020) form a closed population (n = 110). Year A completed the microscopy course (part B) in digital form, year B completed an integrated course with reduced attendance time. At the time of the survey, the participants had already completed their second semester.

Both the evaluation form and the determination course were published via the survey portal of Leipzig University (version 3.27.12, LimeSurvey GmbH, Hamburg, Germany, https://umfrage.uni-leipzig.de/, last accessed: 01.10.2023).

The 98 items of the evaluation questionnaire were divided into six groups: *personal details* (A), *perception of the online semester* (B), *conditions for digital/hybrid teaching* (C), *macroscopic anatomy* (D), *microscopic anatomy* (E) and *future perspectives* (F) (see *Extended data*, (( Winter (2024)). Only selected items from sections A, E and F are relevant for answering the questions in focus here. The personal details were used exclusively to characterize the sample and do not allow any conclusions to be drawn about identifiable participants. In section F, participants assessed the extent to which digital teaching formats should be integrated into their studies in the future. Ordinally scaled items could be evaluated using a five-point verbalized Likert scale, the asymmetry of which also allowed participants to take a neutral position. The evaluation questionnaire also contained qualitative elements in the form of open questions and thus represents a mixed-methods approach ( Johnson *et al*., 2007), which serves to improve understanding of the complex research topic ( Kuckartz, 2014).

**Figure 2.**
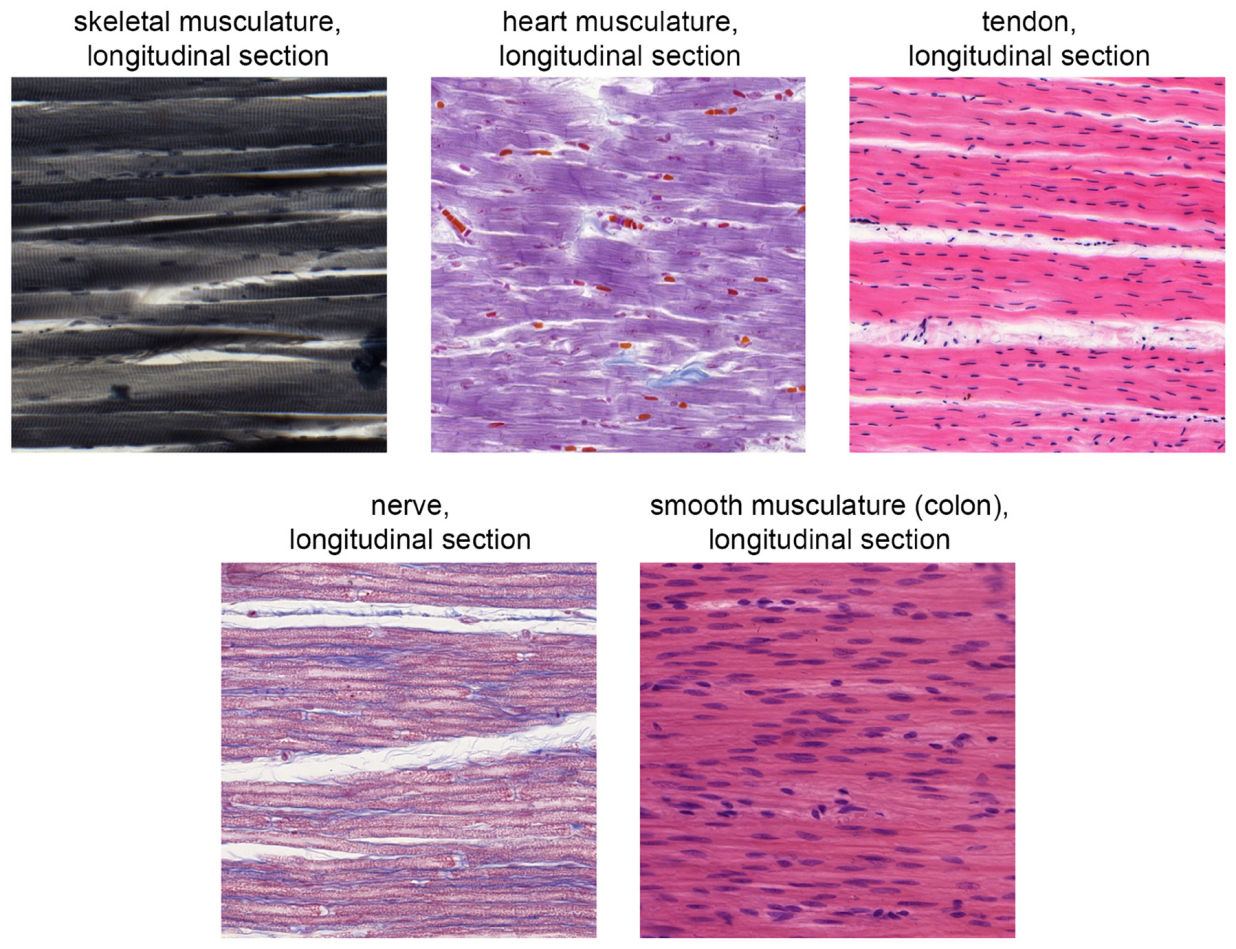
Direct comparison of the five tissue sections shown in the digital identification course from the “musculature, tendon and nerve” group (TG4).

**Histological identification course:** For the histological **identification course**, the students were shown 34 clearly identifiable sections of known tissue sections one after the other and asked to name them using a short word group within 90 seconds. The sequence was based both on the didactic structure of the course already completed and on the classification of easily confusable specimens ( Welsch & Sobotta, 1994). Six tissue groups (TG) were defined: *lymphatic organs* (TG1), *transverse sections* (TG2), *glands* (TG3), *musculature, tendon and nerve* (TG4), *shape similarity* (TG5) and *gastrointestinal tract* (TG6). Figure 2 illustrates the difficulty of delimiting the tissues using the example of the musculature, tendon and nerve group.

### Data protection

A corresponding privacy policy was prepared in consultation with the respective data protection officers of Leipzig University and the Faculty of Medicine. When the study participants accessed the CUVM website and the survey portal of Leipzig University for the first time, all study participants had to accept these data protection conditions.

### Data analysis

The quantitative data was analyzed using Microsoft ® Excel for Mac (Version 16.56, Microsoft Corporation, Redmond, USA) and Wolfram Mathematica (Version 12.2, Wolfram Research Inc., Champaign, IL, USA). The qualitative elements were analyzed in the form of a quantitative content analysis ( Döring *et al*., 2016) using MAXQDA Analytics Pro 2020 (Version: 20.4.2, VERBI GmbH, Berlin). We used the Kruskal-Wallis test ( Rasch *et al*., 2014) to make a statement about the significance of the changes in the results of the determination course between year A and B.

## Results

The sample sizes of the **evaluation questionnaire** varied in a direct comparison of the years and the survey instruments. For example, 59 students from year A and 20 students from year B took part in the evaluation questionnaire. At 72.9% (n = 43) and 90% (n = 18), the proportion of female participants was significantly higher than that of male participants in both rounds. Female students also made up the larger proportion in the selected year groups. The identification course was completed by 37 participants in the first year and 48 in the following year.

At the end of the last section (F) of the evaluation form, participants had the opportunity to express their thoughts, suggestions and criticisms on the topic of digital teaching. One participant from year A considered the approach of “offering the lectures digitally at the same time” to be “a good idea”. This assessment was shared by four other students from both years. At the same time, however, there was criticism of the lack of face-to-face teaching in the practical courses. One participant from year B wrote, for example: “In my opinion, seminars or practical courses cannot be replaced, as there is a lack of direct exchange and, after all, it is about practical knowledge and experience, which can only be acquired with the necessary equipment and in the intended environment.” Four of her fellow students also agreed with this criticism. One student rated the histology course in particular as “really good with the podcasts and the virtual microscope”. Despite this, she also criticized the lack of practice.

These opinions do not stand for themselves, but can be transferred to the population as a whole. The desire for additional or exclusively digital lecture formats was reflected equally in both cohorts: 52.5% (n = 31) of students in cohort A and 75% (n = 15) of students in cohort B were in favor of continuing to offer lectures digitally in the future. Regardless of this, some students, particularly in year A, would prefer a return to face-to-face lectures (A: 18.6%, n = 11; B: 5%, n = 1).

**Figure 3.**
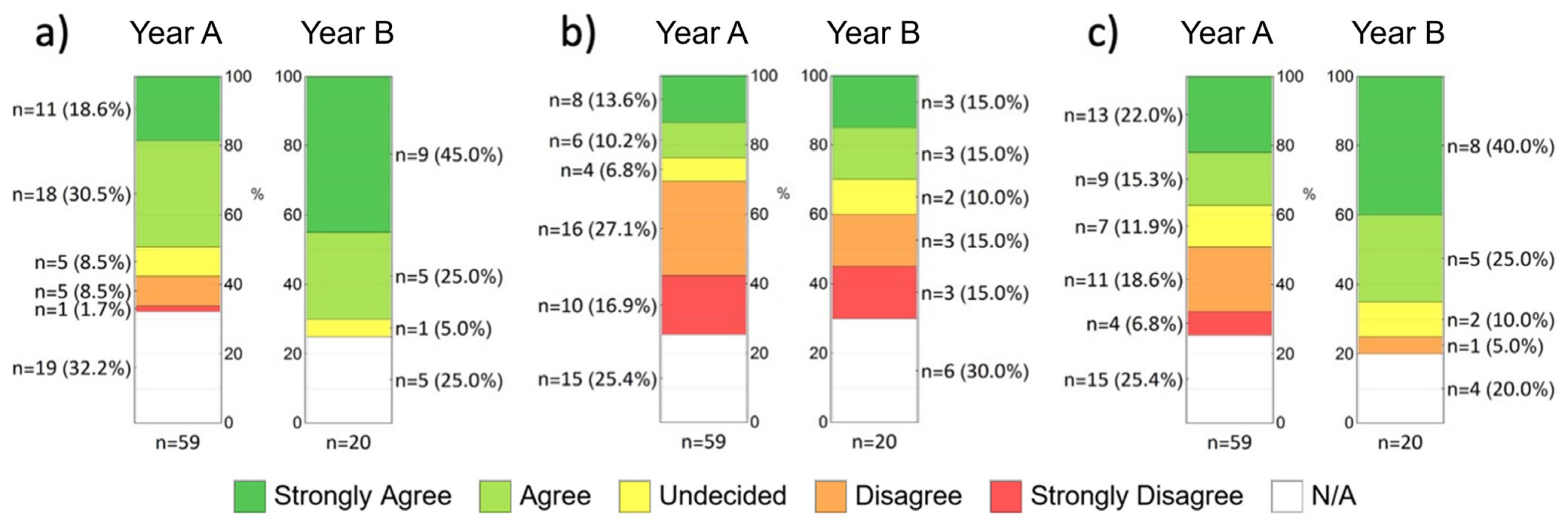
Students' assessment of selected statements (items) from the evaluation form using a five-point Likert scale. **a**) “I was able to adequately acquire the teaching content of microscopic anatomy with the help of the CUVM despite the lack of classroom teaching.”; **b**) “The virtual microscope can replace traditional microscopy in class.”; **c**) “I can imagine an integration of classroom teaching and online studies for the subject of anatomy.”

The participants were also asked to assess whether they were able to adequately acquire the teaching content of microscopic anatomy with the help of the CUVM ( Figure 3a). In percentage terms, more participants in year B selected the top box (strongly agree or somewhat agree) than in year A (A: 49.1 %, n = 29; B: 70 %, n = 4). In contrast, 10.2% of students from year A tended to agree or disagreed with this statement (n = 6). In addition, many participants were against a complete substitution of classroom teaching with the classic light microscope ( Figure 3b). In year A, almost half of the respondents were of the opinion that the CUVM could not replace the conventional light microscope (44 %, n = 26), while 23.8 % (n = 14) disagreed with this statement. In year B, almost a third still shared this opinion (30%, n = 6), with an equally large proportion expressing the opposite view (30%, n = 6). An alternative is the integration of face-to-face teaching and digital teaching ( Figure 3c), which 37.3% (n = 22) from year A and 65% (n = 13) from year B somewhat or fully agreed with. The majority of students used the CUVM as exam preparation (A: 59.3 %, B: 70 %). It was also very popular among respondents as preparation for the microscopy course (A: 40.7 %, n = 24; B: 45 %, n = 9) and to illustrate textbook content (A: 33.9 %, n = 20; B: 40 %, n = 8).

**Figure 4.**
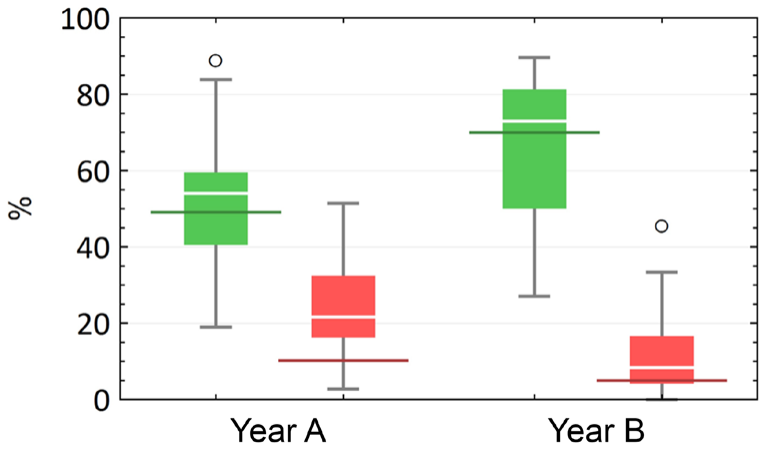
Development of the percentage of correct (green) and incorrect (red) answers in relation to the individual preparations in a comparison of the two cohorts ( **A** and **B**) with each other. In addition, the results from Figure 3a (subjective assessment of learning success) are compared with the objective learning success (red and green horizontal markings).

The results of the **identification course** provide an impression of the actual learning success. The course was deemed to have been passed as soon as a participant had correctly identified at least 60 % or 21 of the specimens sought. In year A, 16 participants (43.2 %) passed the test, in year B it were more than twice as many (n = 34), which corresponds to 70.8 % of the total number of participants. These results correlate with the subjectively perceived learning success of the students ( Figure 4): Year A had slightly overestimated itself in this respect, while year B had assessed itself very realistically. TG 3 (glands) and TG 4 (muscles, tendon and nerve) caused the most problems for students in both years. Looking at the individual tissue sections in terms of the respective number of correct and incorrect answers, clear tendencies can be seen when comparing the year groups. In both rounds, there were tissue sections that were almost exclusively answered correctly or incorrectly.

While the median of correct answers per preparation increased (MdA = 54.1 %, MdB = 72.9 %), the median of incorrect answers decreased (Md _A_ = 21.6 %, Md _B_ = 8.3 %). However, these changes were not significant (p _correct_ = 0.313; p _incorrect_ = 0.138). As a direct consequence, the difference or range between the median of correct and incorrect answers per tissue section increased in year B (∆ _A_ = 32.4 %, ∆ _B_ = 64.6 %). This indicates a significant decrease in incorrect answers in favor of correct answers compared to the previous year (A) (p < 0.001).

## Discussion

The integration of digital teaching formats into (dental) medical curricula was discussed in detail at the latest with the outbreak of the COVID-19 pandemic, and the strengths and weaknesses of online formats were compared ( Barbeau *et al*., 2013; Darici *et al*., 2021; Heidger *et al*., 2002; Maity *et al*., 2023; Vanka *et al*., 2020). Their increased use was predicted as a “central component” of medical education, training and continuing education ( Ruf *et al*., 2008). Last but not least, they are an expression of “modern, contemporary standards in dental training” ( Kahl-Nieke & Vonneilich, 2018). With regard to microscopy, however, the legitimate question is also raised as to whether the digital format can really be described as such, as the skill of operating a microscope is not taught and the variability of the tissue specimens is also lost if the students only ever have one unique digital example ( Schmidt, 2013).

The results suggest that the students surveyed are in favor of integrating digital teaching formats into anatomy teaching in the future. However, a distinction must be made between the year groups surveyed and the various teaching formats. The majority of participants are in favor of retaining digital or hybrid lecture formats. This internal study observation is confirmed by a cross-university survey conducted by the “Stifterverband für die Deutsche Wissenschaft” (donors' association for german science), in which 55% of students rated digital lectures as at least as good as those in the lecture hall ( Wie bewerten Sie die folgenden Lehr- und Prüfungsarten in digitaler Form an Hochschulen?, 2020). Other studies support this trend ( Hempel *et al*., 2022; Stoehr *et al*., 2021). However, the respondents who were still able to complete their first semester in attendance and then had to complete a purely digital summer semester (year A) had a much more divided opinion regarding future *blended learning* approaches than year B, who had been confronted with integrated formats since the beginning of their dental studies. It is only possible to classify these results by taking into account the prevailing framework conditions: at the beginning of the pandemic, Year A was confronted with *emergency remote teaching* (ERT), which, due to its emergency nature (quick, temporary solutions), should by no means be equated with specially developed and long-tested e-learning methods ( Hodges *et al*., 2020). From fall semester 2020 (first semester of year B), a partial return to face-to-face teaching was possible, and some of the problems of the digital semester have already been partially identified and adequately addressed.

With regard to the return to face-to-face teaching in microscopic anatomy courses and the associated lecture, which has been fully implemented since the spring semester of 2022, the discrepancy already observed by Stoehr between students' wishes regarding digital offerings and the prevailing curricula can also be observed here ( Stoehr *et al*., 2021). The legal anchoring of integrated approaches in the new regulations on the licensing of dentists (articles 6 to 9, ZApprO) and the continuously developing digital infrastructure at German universities ( Lübcke *et al*., 2021) underline this contradiction all the more clearly.

A direct comparison of the **subjective and objective learning success** of both cohorts shows that cohort B completed the identification course much more successfully and also assessed themselves more realistically in this respect. Year A, on the other hand, had slightly overestimated itself and achieved poorer results. These data contradict studies that attest to the high learning success of a purely digital histology course ( Studienordnung für den Studiengang Zahnmedizin an der Universität Leipzig, 2022). However, the ERT situation should be taken into account here. While the collected data speaks against a purely digital course despite the CUVM being considered helpful, there are contrasting results for integrated approaches compared to traditional face-to-face teaching ( Ruf *et al*., 2008). The promising potential of *blended learning* for future dental education has already been emphasized many times ( Schmidt, 2013; Wie bewerten Sie die folgenden Lehr- und Prüfungsarten in digitaler Form an Hochschulen?, 2020). In this regard, supporting data are available for microscopic anatomy teaching in particular ( Cheng *et al*., 2017).

Although many authors have already dealt with the detailed evaluation of various facets of anatomical teaching during the COVID-19 pandemic, according to current knowledge, the multi-perspective mixed-methods approach of this study is particularly noteworthy. The central limitation results from the samples examined. It is only possible to compare purely digital ERT and *blended learning* approaches. In view of the current developments back towards pure face-to-face teaching, it would be worthwhile carrying out a new evaluation and integrating the results into the current discourse on contemporary teaching formats.

## Conclusion

The reform efforts of recent years have resulted in the adoption of a new ZApprO, which is intended to fundamentally modernize the study of dentistry in Germany. Triggered by the pandemic-related switch to purely digital teaching and the associated legal hurdles, the demand for a formal legal framework for digitally supported teaching methods was finally met in September 2021. The data collected as part of this evaluation from the digital spring semester 2020 and the integrated spring semester 2021 underline the potential of *blended learning* approaches for dental education. This applies in particular to lecture formats. In addition, practical courses with appropriate resources (e.g. CUVM, digital identification course) can benefit from this approach. The objectively measured learning success of Year B, which completed the microscopy course alternately on site and digitally, also speaks in favor of integrated forms of teaching, despite the potential for improvement that needs to be addressed. This contrasts with the current return of the course to purely face-to-face teaching. The study confirms the already recognized discrepancy between students' preferences and the current status quo ( Stoehr *et al*., 2021). The resulting demand with regard to future-oriented, modern teaching should be to avoid a complete return to pure face-to-face teaching and to strive for integrated didactic methods. As internships under the new ZApprO are always subject to the same principles and objectives (article 7, ZApprO), it is possible to transfer the experience gained to other courses, taking into account the respective prerequisites and framework conditions.

With regard to the training of clinical and practical skills, face-to-face teaching should not and cannot be dispensed with ( Atwa *et al*., 2022). The faculties should primarily evaluate the success of the digital formats they developed as an emergency solution (ERT) at the beginning of the pandemic and further expand the existing potential in a targeted manner on the basis of the knowledge gained. For microscopic anatomy at the Leipzig University, this specifically means further developing the CUVM and the digital identification course. Generally speaking, digital education and training should enable the next generation of healthcare professionals to cope with crises such as the COVID-19 pandemic ( Caruso, 2021; Tolks *et al*., 2020).

## Ethics and consent

We obtained ethical approval (no: 007/21ek) on 19th January 2021, prior to the start of our study from the ethics committee of the Medical Faculty at the University of Leipzig. All study participants were informed in advance about the contents of the study by e-mail and written informed consent to participate was obtained at the start of every individual questionnaire. All work adhered to the Declaration of Helsinki.

## Data Availability

Zenodo: Microscopy without a microscope. https://doi.org/10.5281/zenodo.14525994 ( Winter (2024)). The project contains the following underlying data: Bestimmungskurs 1.0 überarbeitet.xlsx (Data for the first identification course A. Currently only the German version is available. A translated version in English will be provided upon reasonable request via the corresponding author.) Bestimmungskurs 2.0 überarbeitet.xlsx (Data for the second identification course B. Currently only the German version is available. A translated version in English will be provided upon reasonable request via the corresponding author.) Evaluationsbogen 1.0_SPSS-Export.xlsx (Data for the first evaluation questionnaire A. Currently only the German version is available. A translated version in English will be provided upon reasonable request via the corresponding author.) Evaluationsbogen 2.0_SPSS-Export.xlsx (Data for the second evaluation questionnaire B. Currently only the German version is available. A translated version in English will be provided upon reasonable request via the corresponding author.) Zenodo: Microscopy without a microscope. https://doi.org/10.5281/zenodo.14525994 ( Winter (2024)). The project contains the following extended data: Histocourse.pdf (Currently only the German version is available. A translated version in English will be provided upon reasonable request via the corresponding author.) Questionnaire.pdf (Currently only the German version is available. A translated version in English will be provided upon reasonable request via the corresponding author.) Data are available under the terms of the Creative Commons Attribution 4.0 International license (CC-BY 4.0).

## Software availability

Excel (Version 16.56, Microsoft Corporation, Redmond, USA), Mathematica (Version 12.2, Wolfram Research Inc., Champaign, IL, USA) and MAXQDA (Version: 20.4.2, VERBI GmbH, Berlin). Excel was used for data collection (alternative software: LibeOffice, https://libreoffice.org), Mathematica was used for statistics and graphics (alternative: R, https://www.r-project.org/) and MAXQDA (alternative: QualCoder, https://qualcoder.wordpress.com/) was used for quantitative content analysis. We have not implemented any proprietary software or algorithms for this study.
