# Microscopy without a microscope? Opportunities and limitations of dental studies in anatomy during the COVID-19 pandemic in Germany

**DOI:** 10.12688/mep.20580.3

**Published:** 2025-07-28

**Authors:** Ann-Kathrin Peters, Karsten Winter, Elisabeth Witt, Hubert-Mario Kuntzsch, Sabine Löffler

**Affiliations:** 1 Institute of Anatomy, University of Leipzig, Leipzig, Saxony, Germany

**Keywords:** COVID-19 pandemic, regulations on the licensing of dentists, dental studies, virtual microscopy, evaluation

## Abstract

**Aim:**

Due to the outbreak of the COVID-19 pandemic, the microscopy course as part of the dental medical training in Leipzig, Germany, was transformed from a classroom-only course into a purely digital and later hybrid course with reduced attendance time. Aim of this educational research study is the detailed evaluation of digital and hybrid courses regarding students’ perception and learning outcome. Based on the findings current teaching should be critically scrutinised, resulting in a modern design of the histology curricula.

**Methods:**

This is an educational research study. The students’ subjective experiences are measured with the help of an evaluation survey and the learning success was objectively measured using a digital assessment course. Data was collected in two consecutive year groups of dentistry students, each after completion of the second semester. Cohort A consisted of 56 students, cohort B of 54 students. The sampling was purposive and the sample size equals the sample population (closed population of A+B = 110). A statistial analysis of the quantitative results were carried out and quantitative results were analysed using content-structuring qualitative content analysis.

**Results:**

Students are predominantly positive about hybrid teaching approaches, although limitations such as perceived deterioration of the quality of teaching and insufficient learning success in cohort A can be identified. Hybrid teaching cohort B showed significantly better learning success than the purely digital cohort A.

**Discussion:**

Particularly digital lecture formats and the CUVM have been valued by students. In addition, practical courses with appropriate resources (e.g. CUVM, digital identification course) can benefit from a blended approach. Regardless of the continued importance of practical training in dental studies, digital teaching formats developed as an emergency solution at the beginning of the pandemic should be evaluated in regard to their success and the existing potential should be further expanded in a targeted manner.

## Introduction

Over the past 30 years, the teaching of microscopic and macroscopic anatomy has changed significantly ( Chapman *et al.*, 2020; Trelease, 2016). Even before the outbreak of the COVID-19 pandemic, digital media were already used intensively by students ( Friedrich & Persike, 2016). In particular, the development of virtual microscopy (VM) at the end of the 20th century ( Harris *et al.*, 2001) facilitated blended learning approaches. VM uses digitized whole slide images of histological specimen, that can either be integrated into face-to-face teaching ( Krippendorf & Lough, 2005; Mione *et al.*, 2013), used as part of a pure *e-learning* ( Darici *et al.*, 2021) or as part of a *blended learning* approach ( Cheng *et al.*, 2017; Xiao & Adnan, 2022). The latter is a combination of face-to-face and computer-based instruction ( Graham, 2006).

While many universities were already successfully integrating digital teaching methods such as VM into their histology courses, the concept in Leipzig, Germany, however, focused mainly on traditional classroom teaching with a light microscope (LM), using VM only as an additional feature for self-studies and exam preparation at home. This changed rapidly with the outbreak of the pandemic in 2020, when in-class teaching had to be completely or at least partially replaced by digital teaching. Hence there was a new urgency to make greater and different use of already existing digital teaching means and to develop new ones to ensure the continuation of teaching under these difficult circumstances. This shift to digital teaching methods can be summarized by the term **emergency remote teaching** (ERT), which was first coined by Charles Hodges *et al*. and aims to differentiate digital teaching approaches that were quickly developed due to the restrictions caused by the pandemic situation from proven pre-pandemic digital and hybrid teaching approaches ( Hodges *et al.*, 2020).

**Reform of dental education:** Even before the pandemic the use of digital media was described as a core competence, whose “development . . . cannot be expected as a by-product of subject-specific knowledge transfer” but requires “targeted and systematic anchoring in curricula” ( Hochschulforum Digitalisierung, 2016). Calls for teaching with “modern didactic methods” and “new training concepts” ( Kahl-Nieke & Vonneilich, 2018) were raised even before the first version of the new regulations on the licensing of dentists (ZApprO; “Approbationsordnung für Zahnärzte und Zahnärztinnen”) was drafted in 2019. At this point, they were still failing to address the need for a legal anchoring of digital teaching. Only in response to the events of the pandemic this aspect was ultimately included with the amendment of September 22, 2021 ("Bundesgesetzblatt”; BGBl, 2021, part I, number 67, page 4335). Since the end of the pandemic situation lectures may continue to be held online (article 6, section 1, sentence 2, ZapprO) and practical exercises and seminars may be “accompanied by digital teaching formats” (article 7, section 2, sentence 5, ZApprO).

The main objective of this study is to evaluate the digital and hybrid histology course during the pandemic retrospectively and identify chances and limitations of the used teaching methods based on students’ perception and their learning outcome in histology teaching. Considering the complete return to face-to-face teaching of histology at Leipzig University it stands in contrast to the conclusion of a german-wide study on the future of medical education where the authors conclude that that “the digital format of medical education will likely continue beyond the COVID-19 pandemic” ( Hertling *et al.*, 2022). Beyond that, a lot of new digital and hybrid teaching approaches have been developed in the course of ERT ( Darici *et al.*, 2021; Gasmalla *et al.*, 2022; Ishak *et al.*, 2022; Xiao & Adnan, 2022), a legal basis for digital teaching formats has been recently implemented (see above), experiences with digital and hybrid teaching in histology classes have already been successful before the pandemic ( Barbeau *et al.*, 2013; Cheng *et al.*, 2017; Krippendorf & Lough, 2005) and discrepancies between students being in favour of digital teaching formats in contrast to the prevailing curricula have also been observed ( Stoehr *et al.*, 2021). Consequently it needs to be discussed as to whether the status quo contradicts the possibilities that we have at hand today to design appropriate future curricula. Do the findings of this study underline this discrepancy and are we failing to exploit the possible potential of digital teaching methods developed during the pandemic?

## Methods

**Figure 1.**
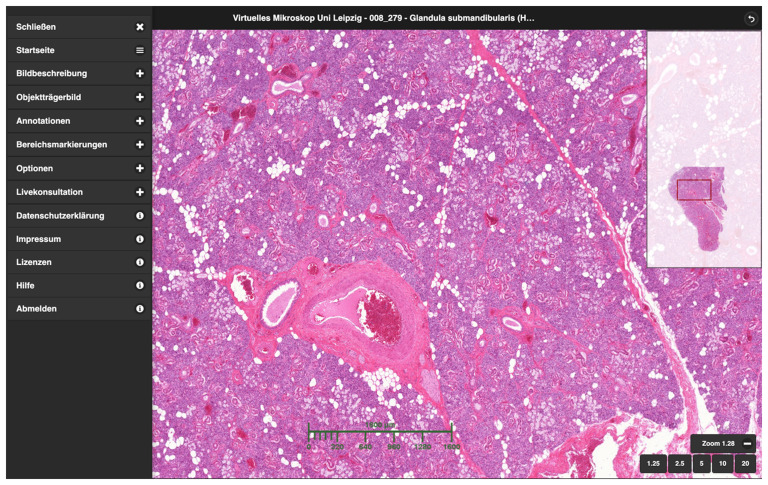
User interface of the CUVM using the example of the submandibular gland. On the left is the fold-out menu, on the right an overview image of the specimen and in the middle the freely navigable digital specimen viewer with a scale bar displayed.

**The microscopy course (pre- and post-pandemic situation):** The study of histology in Leipzig extends over one academic year, divided into two course sections ( Studienordnung für den Studiengang Zahnmedizin an der Universität Leipzig, 2022). Starting with *general histology* in the first semester (part A), students learn the basics for studying *specific organ systems* in the second semester (part B). Theoretical basics are taught in a lecture (on campus) in the first semester. Practical courses are held on campus using a conventional light microscope. Since 2017 students also have additional access to all course specimen via the central e-learning platform *Core Unit Virtual Microscopy* (CUVM, VMscope GmbH, Berlin, Germany) ( Figure 1). Both course sections are completed with a multiple choice exam. Fellow students, who are more advanced in their studies offer support during class (peer-assisted learning). In terms of content, the learning objectives are based on the national competence-based catalogue of learning objectives in dentistry (NKLZ), which defines a “core curriculum” for the subject based on the current regulations on the licensing of dentists ( Nationaler Kompetenzbasierter Lernzielkatalog Zahnmedizin, 2023).

**The microscopy course (during pandemic situation):** In the summer and winter semester 2020, the microscopy course had to be held purely digitally due to ongoing contact restrictions followed by a hybrid summer and winter semester 2021 with reduced attendance times. In the spring semester 2022, there was a complete return to pure face-to-face teaching in its original form (see above). The course content is similar to the in-class histology course. The CUVM fully (2020) respectively partially (2021) replaced LM. In both years lectures were held online, and a digital histological identification course (see below) was developed to enable students` self-assessment and at the same time monitor their learning success at the end of the second semester. Additionally, in the summer semester 2020 a credit-point-system was introduced to encourage student motivation. During the hybrid summer and winter semester 2021 part of the course content was outsourced in form of a flipped-classroom approach: some of the specimen were discussed exclusively during online seminars before the actual in-class teaching with reduced attendance times compared to the initial course. This approach is supposed to allow a more profound discussion in class, since fewer specimen have to be microscoped on site.

To answer the research questions an educational research study was conducted, that aimed at the detailed evaluation of ERT in microscopic anatomy teaching at the Institute of Anatomy at Leipzig University. To take into account as many facets of ERT as possible, quantitative and qualitative elements were used. Thus the study represents a mixed-methods approach ( Johnson *et al.*, 2007), which serves to improve understanding of the complex research topic ( Kuckartz, 2014). The data was collected using an **evaluation survey** and a newly developed **digital histological identification course** (see below). In the evaluation survey aspects such as the digital lecture format, organisation of ERT, student motivation and communication, peer-assisted learning, the use of CUVM and students perceived learning success in the histology course as well as future expectations of the curriculum were examined. Meanwhile the identification course was not only designed to depict students actual learning success but also to serve as a self-assessment tool after course completion.

Data collection took place in two consecutive student cohorts, both of which enrolled in and just finished the first and second semester and therefore part A and B of the histology course. All students enrolled in part B of the course in summer semester 2020 and summer semester 2021 (based on the official lists of the anatomical institute) were included. Within this cohort there were no exclusion criteria. Cohort A (N=56) finished the course in summer 2020, which means they experienced both in-class (first semester) and purely digital teaching (second semester). Cohort B (N=54) finished the course the following year in summer 2021 and therefore experienced purely digital (first semester) and hybrid teaching (second semester). The two cohorts are a closed population (N _A_+N _B_=110), the sampling is purposive, since all participants were chosen intentionally and the sampling size equals the sampling population. Both cohorts had the opportunity to use a LM in one out of two semesters and had access to VM (additionally or exclusively) throughout both semesters.

The different initial situations described above and the special circumstances of the pandemic in general must always be kept in mind, when discussing the results of this study. However, the primary goal is to identify chances and limitations of ERT and not to compare digital and hybrid teaching between the two cohorts.

**Evaluation form:** The 98 items of the survey were divided into six groups: *personal details* (A), *perception of the online semester* (B), *conditions for digital/hybrid teaching* (C), *macroscopic anatomy* (D), *microscopic anatomy* (E) and *future perspectives* (F) (see *Extended data*, ( Winter (2024)). Only selected items from sections A, E and F are relevant for answering the questions in focus here. The personal details do not allow any conclusions to be drawn about identifiable participants. Ordinally scaled items could be evaluated using a five-point verbalized Likert-like scale, the asymmetry of which also allowed participants to take a neutral position. The evaluation questionnaire also contained nominal scaled items (single- and multiple-choice questions) and qualitative elements in the form of open questions.

**Figure 2.**
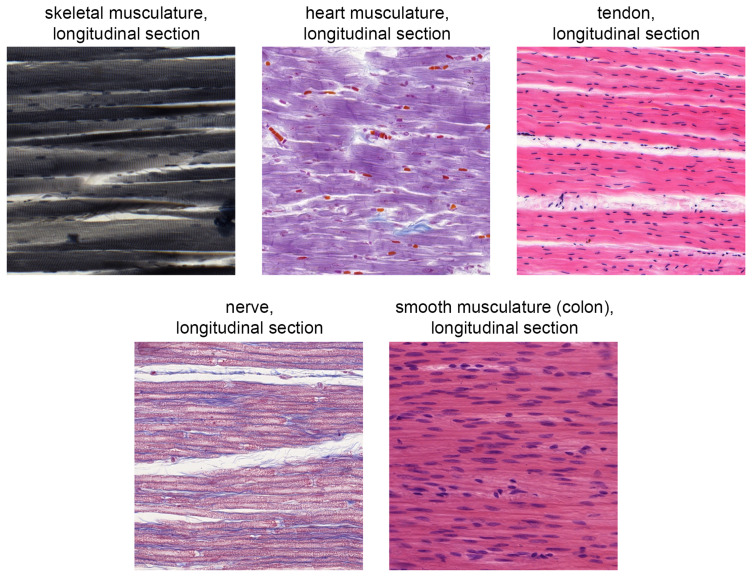
Direct comparison of the five tissue sections shown in the digital identification course from the “musculature, tendon and nerve” group (TG4).

**Digital histological identification course:** The digital histological identification course was developed during the course of ERT to depict student performance and serve as a self-assessment tool for the students. After having completed the second semester and therefore part A and B of the histology course, students were shown 34 clearly identifiable sections of known tissue samples from part A and B of the histology course one after the other and asked to name them within 90 seconds using a short word group. The sequence was partly based on the didactic structure of the course but also focused on the differentiation of easily confusable specimens ( Welsch & Sobotta, 1994). Six tissue groups (TG) were defined: *lymphatic organs* (TG1), *transverse sections* (TG2), *glands* (TG3), *musculature, tendon and nerve* (TG4), *shape similarity* (TG5) and *gastrointestinal tract* (TG6). Figure 2 illustrates the difficulty of delimiting the tissues using the example of TG4.

Both the evaluation form and the determination course were published via the survey portal of Leipzig University (version 3.27.12, LimeSurvey GmbH, Hamburg, Germany, https://umfrage.uni-leipzig.de/, last accessed: 01.10.2023).

### Data protection

A corresponding privacy policy was prepared in consultation with the respective data protection officers of Leipzig University and the Faculty of Medicine. When the study participants accessed the CUVM website and the survey portal of Leipzig University for the first time, all study participants had to accept these data protection conditions.

### Data analysis

The qualitative elements were analysed in the form of a content-structuring qualitative content analysis ( Kuckartz, 2014). using MAXQDA Analytics Pro 2020 (Version: 20.4.2, VERBI GmbH, Berlin). The quantitative data was analysed using Microsoft ® Excel for Mac (Version 16.56, Microsoft Corporation, Redmond, USA), IBM SPSS Statistics (Version 27, IBM Corp., Armonk, New York, USA) and Wolfram Mathematica (Version 12.2, Wolfram Research Inc., Champaign, IL, USA). Descriptive statistic was computed, stacked bar charts for data from Likert scales and boxplots for interval-scaled data were generated. Mann-Whitney-U test was used to test for group differences of students' assessment of selected statements (ordinal scaled Likert items) from the evaluation form. Data from results of the determination course between year A and B (interval-scaled data) was tested for normal distribution using the Shapiro-Wilk test and testing for group differences was performed using the Kruskal-Wallis test ( Rasch *et al.*, 2014) with subsequent Dunn's post-hoc tests (not normally distributed data) to adjust the p-value for multiple group comparisons. Significance for all tests was set at p < 0.05.

## Results

**Participation rates:** 59 out of 56 students (105.4 %) from cohort A and 20 out of 54 students (37 %) from cohort B took part in the **evaluation questionnaire**. The 105.4 % most likely come about due to participants accessing the questionnaire twice via different end devices. The participation rate of the **identification course** was 68.5 % (n _A_ = 37) in cohort A and 88.9 % (n _B_ = 48) in cohort B. While participation rates regarding the identification course were high throughout both years, the low participation rate of cohort B regarding the evaluation questionnaire must be viewed critically.

**Results of the questionnaire:** Percentages refer to the total number of participants for each cohort. Looking at the results of the **general assessment** of the digital and hybrid semester based on Likert-like items revealed that students from cohort A particularly, strongly agree resp. agree with the statement that ERT had a negative impact on the quality of teaching (45.8 %). In cohort B only 30 % (strongly) agree with this statement. At the same time, many students from both cohorts strongly agree resp. agree, that the digitization forced by the pandemic was long overdue (Cohort A: 37.7 %; Cohort B: 50 %) and thus represents a positive development (Cohort A: 40.7 %; Cohort B: 65 %). Especially when it comes to the desired format of future **lectures**, a clear tendency of students' preferences can be observed. The desire for additional or exclusively digital lecture formats is reflected equally in both cohorts: 18.6 % (n _A_ = 11) of the participants from cohort A and 25 % (n _B_ =5) of the participants from cohort B would prefer a purely digital approach for future lectures. Furthermore, 33.9 % (n _A_ = 20) of the participants from cohort A and 50 % (n _B_ = 10) of the participants from cohort B prefer a blended approach. Especially being able to pause or fast-forward the provided screencast was considered helpful by the students (74.6 % of the students from cohort A and 75 % from cohort B (strongly) agree).

By analysing the qualitative elements of the evaluation form, however, there was criticism of the lack of face-to-face teaching in the practical courses. One participant from year B wrote: “In my opinion, seminars or practical courses cannot be replaced, as there is a lack of direct exchange and, after all, it is about practical knowledge and experience, which can only be acquired with the necessary equipment and in the intended environment.” Four of her fellow students express similar opinions in this regard.

**Figure 3.**
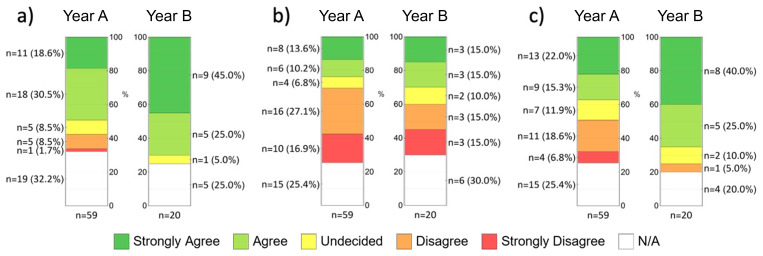
Students' assessment of selected statements (items) from the evaluation form using a five-point Likert scale. Absolute/relative results and p-values for group differences: **a**) “I was able to adequately acquire the teaching content of microscopic anatomy with the help of the CUVM despite the lack of classroom teaching.” (p=0.016); **b**) “The virtual microscope can replace traditional microscopy in class.” (p=0.500); **c**) “I can imagine an integration of classroom teaching and online studies for the subject of anatomy.” (p=0.028)

The participants were also asked to assess their experiences with the **CUVM** with the help of a Likert-like scale. 49.1 % (18.6 % + 30.5 %; n _A_ = 29) of the participants from cohort A and 75 % (45.0 % + 25.0 %; n _B_ = 14) from cohort B (strongly) agree that they were able to adequately acquire the teaching content of microscopic anatomy with the help of the CUVM despite the lack of classroom teaching. In contrast, only 10.2 % (8.5 % + 1.7 %; n _A_ = 6) of students from cohort A and none from cohort B (strongly) disagreed with this statement. There was a significant difference between answers from cohort A and cohort B (p=0.016) ( Figure 3a). Despite the positive assessment of VM, 44 % (27.1 % + 16.9 %; n _A_ = 26) of the participants from cohort A (strongly) disagree that VM can replace LM in class, whereas in cohort B just as many students agree with the statement (each: 30 %; 15.0 % + 15.0 %; n _B_ = 6) as disagree with it (no significant difference between the two cohorts; p=0.500). ( Figure 3b). Considering the possibility of hybrid anatomy teaching, 37.3 % (22.0 % +15.3 %; n _A_ = 22) of the participants from cohort A and 65 % (40.0 % + 25.0 %; n _B_ = 13) from cohort B (strongly) agree that they can imagine an integration of digital and face-to-face teaching, whereas 25.4 % (18.6 % + 6.8 %; n _A_ = 15) of the participants from cohort A and only one person from cohort B (5.0 %) (strongly) disagree with this statement. There was a significant difference between answers from cohort A and cohort B (p=0.028) ( Figure 3c). Moreover, the participants were asked to name the advantages and disadvantages of the CUVM in a free text. Qualitative analysis of the answers with QDA software shows that being able to use VM for self-studies and exam preparation is a central advantage, whereas missing annotations in the software, the optimized and therefore unrealistic presentation of the digital glass slides and the lack of variability are central disadvantages.

**Figure 4.**
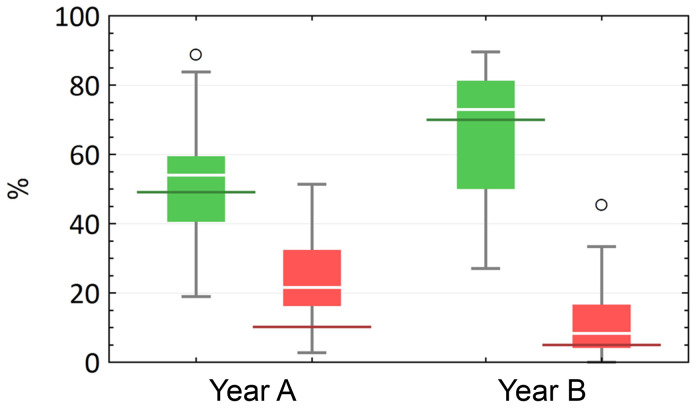
Development of the percentage of correct (green) and incorrect (red) answers in relation to the individual preparations in a comparison of the two cohorts ( **A** and **B**) with each other. For each year there were significant differences between correct (p<0.001) and incorrect (p<0.001) answers, but there were no significant differences for the increase/decrease of correct/incorrect answers between year **A** and year **B** (p=0.313 and p=0.138, respectively). In addition, the results from Figure 3a (subjective assessment of learning success) are compared with the objective learning success (red and green horizontal markings).

**Results of the identification course:** The course was considered passed as soon as a participant had correctly identified at least 60 % resp. 21 of the specimen sought. In cohort A, a total of 16 out of 37 (43.2 %) students passed the identification course. In year B, however, there were more than twice as many students that passed (n _B_ = 34), which corresponds to 70.8 % of the total number of participants. These results correlate with the subjectively perceived learning success of the students ( Figure 4): Cohort A had slightly overestimated itself in this respect, while year B had assessed itself realistically. TG 3 (glands) and TG 4 (muscles, tendon and nerve) caused the most problems for students in both years. Looking at the individual tissue sections in terms of the respective number of correct and incorrect answers, clear tendencies can be seen when comparing the year groups. In both rounds, there were tissue sections that were almost exclusively answered correctly or incorrectly.

Statistical analysis showed that for each year there were significant differences between correct (p<0.001) and incorrect (p<0.001) answers with an increase of correct/incorrect median differences from 32.4 % (year A) to 64.6 % (year B). From year A to year B the median of correct answers per preparation increased (Md _A_ = 54.1 %, Md _B_ = 72.9 %) not significantly (p=0.313) while the median of incorrect answers decreased (Md _A_ = 21.6 %, Md _B_ = 8.3 %) also without significance (p=0.138). Descriptive statistics are shown in Table 1.

**Table 1.** Descriptive statistics of correct and incorrect answers from cohorts of years A and B.

	Year A		Year B	
	correct	incorrect	correct	incorrect
minimum	18.9 %	2.7 %	27.1 %	0.0 %
maximum	89.2 %	51.4%	89.6 %	45.8 %
median	54.1 %	21.6 %	72.9 %	8.3 %
interquartile range	18.9 %	16.2 %	31.3 %	12.5 %

## Discussion

The integration of digital teaching formats into (dental) medical curricula was discussed in detail at the latest with the outbreak of the COVID-19 pandemic, and the strengths and weaknesses of online formats were compared ( Barbeau *et al.*, 2013; Bianchi *et al.*, 2020; Darici *et al.*, 2021; Gerardi *et al.*, 2024; Heidger *et al.*, 2002; Maity *et al.*, 2023; Vanka *et al.*, 2020; Varvara *et al.*, 2021). Their increased use was predicted as a “central component” of medical education, training and continuing education ( Ruf *et al.*, 2008). Ultimately, they are considered an expression of “modern, contemporary standards in dental training” ( Kahl-Nieke & Vonneilich, 2018).

When discussing and comparing the results of cohort A and B it is important to keep in mind that both cohorts experienced different teaching formats (A: in-class followed by purely digital, B: purely digital followed by hybrid) and therefore compare their experiences to different initial situations. Both the results of the evaluation questionnaire and the histological determination course reveal limitations of ERT, such as a subjective decrease in teaching quality, insufficient learning success or a lack of direct exchange and practical experience. However, the desire for digitisation exists despite these limitations and therefore underlines the deviations between students' wishes and the prevailing curricula, that has already been recognised by other authors ( Stoehr *et al.*, 2021). Students surveyed are in favor of integrating digital teaching formats into histology teaching but at the same time consider obtaining face-to-face teaching important ( Atwa *et al.*, 2022). Otherwise the essential skill of operating a microscope and dealing with a variability of tissue specimen is lost if the students only use VM and therefore have one unique digital example ( Schmidt, 2013). The majority of the participants throughout both cohorts are in favor of retaining digital or hybrid lecture formats. This internal study observation is confirmed by a cross-university survey conducted by the “Stifterverband für die Deutsche Wissenschaft” (donors' association for German science), in which 55 % of the students rated digital lectures as at least as good as those on site ( Wie bewerten Sie die folgenden Lehr- und Prüfungsarten in digitaler Form an Hochschulen?, 2020). Further studies support this trend ( Hempel *et al.*, 2022; Stoehr *et al.*, 2021). Both cohorts rate their work with the CUVM positively and can imagine blended learning approaches in the future. The success of VM regarding students’ satisfaction and learning success was also rated positively by many authors even before the pandemic (( Barbeau *et al.*, 2013; Krippendorf & Lough, 2005; Mione *et al.*, 2013)). However, the respondents from cohort B, who had been confronted with purely digital and integrated formats since the beginning of their dental studies were more in favor of future *blended learning* approaches than cohort A, who were still able to complete their first semester in attendance and then had to complete a purely digital summer semester. It is only possible to classify these results by taking into account the prevailing framework conditions: at the beginning of the pandemic, Year A was confronted with *emergency remote teaching* (ERT), which, due to its nature (quick, temporary solutions), should by no means be equated with specially developed and long-tested e-learning methods ( Hodges *et al.*, 2020). For cohort B a partial return to face-to-face teaching was possible, and some of the problems of the digital semester had already been identified and adequately addressed.

A direct comparison of the **subjective and objective learning success** of both cohorts shows that cohort B completed the identification course much more successfully and moreover assessed themselves more realistically in this respect. Students’ self-assessment regarding learning success, however, isn’t necessarily congruent with the actual learning outcome. The poor learning success of cohort A contradicts studies that attest to the high learning success of a purely digital histology course ( Barbeau *et al.*, 2013; Darici *et al.*, 2021). But again, the ERT situation must be taken into account here. The promising potential of *blended learning* for future dental education has already been emphasized ( Schmidt, 2013; Wie bewerten Sie die folgenden Lehr- und Prüfungsarten in digitaler Form an Hochschulen?, 2020). In this regard, supporting data are available for microscopic anatomy teaching in particular ( Cheng *et al.*, 2017).

To summarize the discussed results, students` opinion on ERT and especially VM, their preferences regarding future curricula as well as their learning outcome reveal a significant potential of hybrid teaching methods for histology teaching in the future. If one also takes into account that digital teaching formats are now legally anchored in the new ZApprO, that there’s continuing improvement of digital infrastructure at german universities ( Lübcke *et al.*, 2021) and that the current state of research confirms the potential of hybrid teaching methods, the assumed discrepancy between the possibilities we have today and the status quo in Leipzig (return to face-to-face teaching) becomes even more obvious. Currently we are failing to exploit the potential of the digital teaching methods that were used and developed during the pandemic. Integrating the CUVM more actively into the course (e.g in the form of a flipped-classroom approach) and using the findings to improve and include the histological identification course in future curricula is the first step towards a more flexible curriculum. In addition, this would make it possible to react more quickly and adequately to similar situations in future. Nonetheless, it is of utmost importance when integrating ERT based methods into high quality curricula in the long term that this implementation is accompanied by constant improvement and evaluation, also taking into account the instructor`s perspective, as well as financial and human resources. Generally speaking, digital education and training should enable the next generation of healthcare professionals to cope with crises such as the COVID-19 pandemic ( Caruso, 2021; Tolks *et al.*, 2020).

**Limitations of the study:** Although many authors have already dealt with the detailed evaluation of various facets of anatomical teaching during the COVID-19 pandemic, according to current knowledge, the multi-perspective mixed-methods approach of this study is particularly noteworthy. Due to the very changeable and hardly predictable framework conditions at the time the study was carried out, as well as the limited time and resources for the development of adequate ERT methods, there are inevitable limitations to this study. The focus was primarily on the student perspective. However, the experiences of teachers and tutors can also provide an important impetus for the evaluation and improvement of digital and hybrid teaching. Moreover, there is no clear distinction between the two cohorts concerning the mode of teaching, since both groups experienced different stages of the pandemic and therefore a mix of in-class, digital and hybrid teaching. Thus the aim of this study is to identify chances and limitations of the ERT methods and not to compare digital to hybrid teaching in itself. With regards to the evaluation form, a low participation rate in cohort B and a large number of respondents in both cohorts, who chose the neutral position on the likert-like scale, is noticeable. This must be taken into account when evaluating and categorising the results. One possible cause could be the very extensive questionnaire due to the joint evaluation of macroscopic and microscopic anatomy. However, as the results of the questionnaire are considered together with the data from the assessment course, conclusions can nevertheless be drawn regarding the success of ERT.

## Conclusion

The results of this study confirm and underline the already assumed discrepancy between the status quo of histological teaching at Leipzig University and the possibilities we have at hand. Integrating digital teaching methods is widely accepted by students, not only at Leipzig University and even though limitations of the ERT methods can definitely be identified (subjective decrease in teaching quality, insufficient learning success, a lack of direct exchange and practical experience) and must be taken into account, they made it possible to continue teaching during the pandemic and therefore can be considered successful under the given circumstances. Particularly digital lecture formats and the CUVM have been valued by students. In addition, practical courses with appropriate resources (e.g. CUVM, digital identification course) can benefit from a blended approach. By returning to a conventional in-class teaching without evaluating the ERT we’re failing to exploit the potential that the effort of creating digital and hybrid teaching methods during the pandemic offers. Successful analog teaching strategies that have shaped the study of medicine and dentistry to date should not be replaced, but anatomical teaching should be enriched by a complete and well thought-out integration of the digital systems available today to create a sustainable hybrid microscopic-anatomical curriculum. Such an approach must be accompanied by ongoing evaluations and corresponding improvements in every case.

## Ethics and consent

We obtained ethical approval (no: 007/21ek) on 19th January 2021, prior to the start of our study from the ethics committee of the Medical Faculty at the University of Leipzig. All study participants were informed in advance about the contents of the study by e-mail and written informed consent to participate was obtained at the start of every individual questionnaire. All work adhered to the Declaration of Helsinki.

## Data Availability

Zenodo: Microscopy without a microscope. https://doi.org/10.5281/zenodo.14793011 ( Winter (2024)). The project contains the following underlying data: Bestimmungskurs 1.0 überarbeitet.xlsx (Data for the first identification course A. Currently only the German version is available. A translated version in English will be provided upon reasonable request via the corresponding author.) Bestimmungskurs 2.0 überarbeitet.xlsx (Data for the second identification course B. Currently only the German version is available. A translated version in English will be provided upon reasonable request via the corresponding author.) Evaluationsbogen 1.0_SPSS-Export.xlsx (Data for the first evaluation questionnaire A. Currently only the German version is available. A translated version in English will be provided upon reasonable request via the corresponding author.) Evaluationsbogen 2.0_SPSS-Export.xlsx (Data for the second evaluation questionnaire B. Currently only the German version is available. A translated version in English will be provided upon reasonable request via the corresponding author.) Zenodo: Microscopy without a microscope. https://doi.org/10.5281/zenodo.14793011 ( Winter (2024)). The project contains the following extended data: histocourse.pdf histocourse_ENGLISH.pdf questionnaire.pdf questionnaire_ENGLISH.pdf Data are available under the terms of the Creative Commons Attribution 4.0 International license (CC-BY 4.0).

## Software availability

Excel (Version 16.56, Microsoft Corporation, Redmond, USA), Mathematica (Version 12.2, Wolfram Research Inc., Champaign, IL, USA) and MAXQDA (Version: 20.4.2, VERBI GmbH, Berlin). Excel was used for data collection (alternative software: LibeOffice, https://libreoffice.org), Mathematica was used for statistics and graphics (alternative: R, https://www.r-project.org/) and MAXQDA (alternative: QualCoder, https://qualcoder.wordpress.com/) was used for quantitative content analysis. We have not implemented any proprietary software or algorithms for this study.
