# Supplementary material for: Microscopy without a microscope? Opportunities and limitations of dental studies in anatomy during the COVID-19 pandemic in Germany
Source: MedEdPublish (2016). 2025 Jul 28;15:2. Originally published 2025 Jan 9. [Version 3] doi: 10.12688/mep.20580.3 (PMC12329405; doi:10.12688/mep.20580.3)
Supplement: Peer review history

# Reviewer report: Reviewer response for version 3

**DOI:** 10.21956/mep.22639.r42564

**Published:** 2025-08-06

**Authors:** Wita Anggraini

**Affiliations:** 1 Universitas Trisakti, Jakarta, Indonesia

Dear Authors

 Congratulations on your excellent writing.

 I am satisfied with the improvements you have made. I suggest you check the Results Section, paragraph 4, for n in group B; where in version 2 it was 4, while in version 3, n=14.

 Congratulations and good luck to all of you.

Have any limitations of the research been acknowledged?

Partly

Is the work clearly and accurately presented and does it cite the current literature?

No

If applicable, is the statistical analysis and its interpretation appropriate?

Partly

Are all the source data underlying the results available to ensure full reproducibility?

No

Is the study design appropriate and is the work technically sound?

No

Are the conclusions drawn adequately supported by the results?

Partly

Are sufficient details of methods and analysis provided to allow replication by others?

Partly

Reviewer Expertise:

Human Anatomy and Histology; Ergonomic Dentistry; Gerodontology and Periodontology

I confirm that I have read this submission and believe that I have an appropriate level of expertise to confirm that it is of an acceptable scientific standard.

# Reviewer report: Reviewer response for version 3

**DOI:** 10.21956/mep.22639.r42563

**Published:** 2025-08-06

**Authors:** Sara Bernardi

**Affiliations:** 1 University of L’Aquila, L’Aquila, Italy

manuscript can be now accepted

Have any limitations of the research been acknowledged?

Partly

Is the work clearly and accurately presented and does it cite the current literature?

Partly

If applicable, is the statistical analysis and its interpretation appropriate?

Yes

Are all the source data underlying the results available to ensure full reproducibility?

Yes

Is the study design appropriate and is the work technically sound?

Yes

Are the conclusions drawn adequately supported by the results?

Yes

Are sufficient details of methods and analysis provided to allow replication by others?

Yes

Reviewer Expertise:

anatomy

I confirm that I have read this submission and believe that I have an appropriate level of expertise to confirm that it is of an acceptable scientific standard.

# Reviewer report: Reviewer response for version 2

**DOI:** 10.21956/mep.22441.r41668

**Published:** 2025-05-29

**Authors:** Wita Anggraini

**Affiliations:** 1 Universitas Trisakti, Jakarta, Indonesia

**Review Results and Suggestions**

 Abstract:

- In the Method, include: type of research, sample population, sampling method (random, consecutive, purposing etc.), subject criteria and formula for determining sample size. Also include the statistical analysis used.
- In the Results, include: the results of statistical analysis, both univariate and bivariate analysis, along with their meaning.
- In the abstract, the Discussion section should briefly focus on the advantages and disadvantages of digital, conventional and hybrid learning methods.
- Include the conclusions of the research in your abstract

Introduction

- Paragraph 2: Explain the purpose of punctuation with a period in brackets.

Methods

- In the Method, include: type of research, sample population, sampling method (random, consecutive, purposing etc.), subject criteria (exclusion and inclusion)   and formula for determining sample size.

Results

- Paragraph 1:
  - Write your sample size clearly, why you use the word "for example"  this gives the impression that the research subjects did not do their job accurately.
  - Explain your sample size based on the statistical formula used. Based on your writing in the Results section, it is not clear what the purpose is and why you are presenting these numbers, such as: 72.9% (n=43) and 90% (n=18)
  - lines 8-10: This statement is difficult to understand. If you want to explain the research results in a narrative, you must explain the chronology of the numbers you listed related to the number of research subjects.
- Paragraph 3, lines 4-8:
  - Group A= 52.5% + 18.6% = 71.1% (n= 31+11=42)
  - Group B= 75% + 5%= 80% (n=15+1=16)
  - The numbers above cannot explain the number of students who are the subjects of the research and there is no explanation at 100%
  - Please convey this narrative in more detail and focus more on what you have measured.
- Paragraph 4, lines 6,8, 12,13,14,15
  - Same with my previous comment above, the numbers and percentages in the results are not clear.
  - Whereas in Figure 3, the number of samples and the recapitulation of answers in percentages and the number n, are very clear.
  - Point of view in the Results section, all questionnaire questions are presented and the results are listed in a descriptive statistical analysis table (minimum, maximum, median, mean, standard deviation) followed by the results of the bivariate statistical analysis (Kruskal Wallis test). So that the research results are easy for readers to understand

Discussion

- Basically, histology learning is learning through observation on histology slides. This means that this histology course can indeed be taught online. The strength of teaching lies in the teaching method of the lecturer, so that students first understand the macro anatomy (for example, what organs are cut and made into histology slides, how the cutting direction is according to what is in the histology slide, etc.). The role of the lecturer is very important.
- Why in the discussion, you do not review how the lecturer's efforts to navigate their students to understand the tissue being observed on the histology slide both directly and digitally.
- My suggestion is to include various journals that explain that the current methods of teaching macro and micro anatomy are greatly assisted by digital teaching such as via YouTube etc.
- The limitations of this research are not stated clearly and openly.

Conclusion

- In the conclusion, what you convey in this manuscript should be included in the discussion.
- Make a short and clear conclusion in answering the research problem.

Have any limitations of the research been acknowledged?

Partly

Is the work clearly and accurately presented and does it cite the current literature?

No

If applicable, is the statistical analysis and its interpretation appropriate?

Partly

Are all the source data underlying the results available to ensure full reproducibility?

No

Is the study design appropriate and is the work technically sound?

No

Are the conclusions drawn adequately supported by the results?

Partly

Are sufficient details of methods and analysis provided to allow replication by others?

Partly

Reviewer Expertise:

Human Anatomy and Histology; Ergonomic Dentistry; Gerodontology and Periodontology

I confirm that I have read this submission and believe that I have an appropriate level of expertise to confirm that it is of an acceptable scientific standard, however I have significant reservations, as outlined above.

# Reviewer report: Reviewer response for version 2

**DOI:** 10.21956/mep.22441.r41217

**Published:** 2025-03-27

**Authors:** Erik Idrus

**Affiliations:** 1 lecturer in Oral Biology, University of Indonesia, Depok, West Java, Indonesia

This manuscript presents a study that aims to examine the advantages and disadvantages of histology learning methods, specifically full offline (face-to-face learning), full online (e-learning), and a combination of both systems (hybrid learning).

 The research sample consisted of students from different academic levels (Year A and Year B). Year A students experienced face-to-face learning in Part A (1st semester) and transitioned to e-learning in Part B (2nd semester). Year B students participated in hybrid learning for both Part A and Part B.

 It is of significant interest to analyze the differences among these various learning methods across different student cohorts and to determine how these methods impact evaluation scores at the end of the course.

 In this study, several points should be noted to enhance reader comprehension and mitigate potential biases in the research findings.

- Lack of Clear Research Objectives: The authors did not explicitly define the primary objective of this study. It remains unclear whether the study aimed to assess student satisfaction with the learning methods or to evaluate the effectiveness of these methods in relation to student comprehension of the material.
- Heterogeneous Sample Groups: The sample comprised students from Year A and Year B, who had distinct learning experiences. Year A students experienced face-to-face learning in Part A and transitioned to full online learning in Part B. This disparity allowed Year A students to offer more informed opinions regarding the advantages of each learning method. Conversely, Year B students only experienced hybrid learning in both Part A and Part B. Consequently, comparing subjective data, such as student opinions and perspectives (data from Figure 3), between these two sample populations (Year A and Year B) is challenging.
- Potential Bias in Figure 3 Data: Figure 3 allowed respondents to omit answers for three questionnaire items (N/A answers). The substantial number of respondents who did not provide opinions on these three items may introduce significant bias into the data.
- Insufficient Analysis in Figure 4: Figure 4 did not conduct a thorough analysis of the effectiveness of the learning methods across the Year A and Year B student populations, specifically failing to determine whether significant differences existed.
- Irrelevant Figures 1 and 2: Figures 1 and 2 did not provide information relevant to the research problem and objectives. Therefore, it is recommended that they not be included as primary data.

Considering the extensive body of research examining the effectiveness of various teaching methods and their delivery, this study aims to provide a more specific perspective on histology education within dental studies. This study presents numerous avenues for exploration regarding histology instruction for dental students, with the potential to enhance the overall quality of education.

 In summary, this manuscript has the potential to offer novel insights into effective and efficient histology teaching in dental schools. However, due to several limitations related to the sample (sample size and selection criteria) and the potential bias within the questionnaire content, a major revision of the aforementioned aspects is strongly recommended.

Have any limitations of the research been acknowledged?

Partly

Is the work clearly and accurately presented and does it cite the current literature?

Partly

If applicable, is the statistical analysis and its interpretation appropriate?

I cannot comment. A qualified statistician is required.

Are all the source data underlying the results available to ensure full reproducibility?

Partly

Is the study design appropriate and is the work technically sound?

Partly

Are the conclusions drawn adequately supported by the results?

Partly

Are sufficient details of methods and analysis provided to allow replication by others?

No

Reviewer Expertise:

dental education, biomolecules and histology, hard tissue

I confirm that I have read this submission and believe that I have an appropriate level of expertise to confirm that it is of an acceptable scientific standard, however I have significant reservations, as outlined above.

# Reviewer report: Reviewer response for version 2

**DOI:** 10.21956/mep.22441.r41169

**Published:** 2025-03-13

**Authors:** Michael Hortsch

**Affiliations:** 1 Cell and Developmental Biology, University of Michigan Michigan Medicine (Ringgold ID: 21614), Ann Arbor, Michigan, USA

This manuscript investigates adaptations to histology teaching for dental students at Leipzig University during the COVID-19 pandemic.

 Two groups of dental students were compared, one receiving histology laboratory training entirely online (Year A). However, a clear description how the second group of students (Year B) were instructed is not provided. Were the second group instructed in a blended approach? Did the two groups differ in their access and use of digital microscopy or light microscopes? A confusing description of the course and the two groups of students is part of the introduction. This should not be part of the introduction, but part of the M&M section.

 Virtual histology has been used for a long time (at least 20 years) for the histology education of biomedical students, including dental students. That is very well documented in the published literature. At the reviewer’s institution, online-only histology laboratory instruction for dental students has been used for almost 20 years. It remains unclear whether the use of virtual microscopy was investigated in this manuscript. The title of the paper suggests as much. How were students in Year B instructed?

 How many students were invited each year to participate? Is n=110 for each year or both years together? Please state the participation rate(s). If only 20 out of 110 students participated for Year B, the participation rate is concerningly low and that needs to be addressed.

 I give the authors credit that they went beyond asking students about their opinions about the use of virtual microscopy and included real learning outcome data. As shown in Fig 4, self-assessment of students is not a reliable reflection of actual learning success.

 More as a comment, the assessment test is rather disappointing, students only being asked to identify tissues in light micrographs. That may not be the fault of the authors if the guidelines do not require higher levels of learning. Tissue identification is not a high bar for histology learning. What is puzzling, the learning outcome supposedly was done for the second half of the course, which deals with organs and organ systems. However, Figure 3 shows basic tissue examples, which represent the first part of the histology course.

 What is the ”Histology Identification Course”? Does that describe the normal assessment of dental students learning histology at the University of Leipzig? Or is this a separate test? How does it relate to the lecture and laboratory histology instruction?

 A statistical analysis of the data in Figure 3 is missing. Are any of these differences between Year A and B statistically significant?

 The topic of the guidelines for the histology education of German dental students may be of national importance and interest, but I am less sure that it will be of interest to readers outside of Germany. It may be an important point of discussion, but a more local/national publication would be a better place for this discussion.

 Also, the statistical analysis of the learning outcome is confusing (Figure 4). As only two groups are compared, the Kruskal Wallis test may not be the best test to determine whether the two groups are statistically different or not. Have the authors tested for normal distribution? If the data are normally distributed, a T-test or ANOVA may be more appropriate. Also, learning outcomes are influenced by many factors. Have other variables been excluded? The description of the results is again very confusing. Are the learning outcome between Year A and B statistically significantly different or not?

 A limitations of the study paragraph is missing.

 In summary, the conclusion that a blended teaching approach, which supplements traditional teaching approaches with online learning resources, is well received by learners and favored by instructors is not new and has been proposed by many authors. The description of the two versions of their histology course is incomplete and confusing. Important information is missing. The authors should spell out more clearly, what is compared and what new insights can be gained from their study. Is this study about light microscopes versus virtual microscopy or about online histology learning versus in-person instruction/blended learning? Limitations of the study and confounding variables need to be discussed.

Have any limitations of the research been acknowledged?

Partly

Is the work clearly and accurately presented and does it cite the current literature?

No

If applicable, is the statistical analysis and its interpretation appropriate?

I cannot comment. A qualified statistician is required.

Are all the source data underlying the results available to ensure full reproducibility?

Partly

Is the study design appropriate and is the work technically sound?

Partly

Are the conclusions drawn adequately supported by the results?

No

Are sufficient details of methods and analysis provided to allow replication by others?

No

Reviewer Expertise:

Medical education, e-learning, histology,

I confirm that I have read this submission and believe that I have an appropriate level of expertise to state that I do not consider it to be of an acceptable scientific standard, for reasons outlined above.

# Reviewer report: Reviewer response for version 1

**DOI:** 10.21956/mep.22027.r40892

**Published:** 2025-01-28

**Authors:** Sara Bernardi

**Affiliations:** 1 University of L’Aquila, L’Aquila, Italy

Dear Authors, 

 Congratulations for the study. 

 Pandemic effect on the education are still to see in the future and represented a challenge for both students and lecturers. 

 What is missing in your article is the questionnaire. (i accessed to the website provided but it is in German language. You should provide an English translated version)

 and in the discussion the learning theories which characterized the medical and dental degree, as well as the digital system now available should be fully integrated but no replace the other teaching strategies (see the provided literature)

Have any limitations of the research been acknowledged?

Partly

Is the work clearly and accurately presented and does it cite the current literature?

Partly

If applicable, is the statistical analysis and its interpretation appropriate?

Yes

Are all the source data underlying the results available to ensure full reproducibility?

Yes

Is the study design appropriate and is the work technically sound?

Yes

Are the conclusions drawn adequately supported by the results?

Yes

Are sufficient details of methods and analysis provided to allow replication by others?

Yes

Reviewer Expertise:

anatomy innovation technology in anatomy teaching

I confirm that I have read this submission and believe that I have an appropriate level of expertise to confirm that it is of an acceptable scientific standard, however I have significant reservations, as outlined above.

## References

[1] : Dental Education Challenges during the COVID-19 Pandemic Period in Italy: Undergraduate Student Feedback, Future Perspectives, and the Needs of Teaching Strategies for Professional Development. *Healthcare (Basel)* .2021;9(4) : 10.3390/healthcare9040454 10.3390/healthcare9040454 33921516 PMC8069889

[2] : Evaluation of Effectiveness of Digital Technologies During Anatomy Learning in Nursing School. *Applied Sciences* .2020;10(7) : 10.3390/app10072357 10.3390/app10072357

[3] Gerardi, D., Bernardi, S., Meta, E., Bianchi, S., & Macchiarelli, G. (2024). The role of innovation technology in teaching and learning strategies in anatomy curricula in dental hygiene school. Italian Journal of Anatomy and Embryology, 128(1), 117–124. https://doi.org/10.36253/ijae-15369.

# Response

**Published:** 2025-01-30

**Authors:** Karsten Winter

**Affiliations:** University of Leipzig, Germany

Thank you for reviewing our manuscript!

 We apologize for not having presented the English translation of our questionnaire earlier.

 The translation is now complete and was uploaded to the repository at: 10.5281/zenodo.14774144

 Thank you for providing the relevant literature. We have added this topic and the references to the discussion section.

# Response

**Published:** 2025-02-18

**Authors:** Karsten Winter

**Affiliations:** University of Leipzig, Germany

Thank you for reviewing our manuscript!

 We apologize for not having presented the English translation of our questionnaire as well as the histoscores earlier.

 The translations are now complete and were uploaded to the repository at: 10.5281/zenodo.14793011

 Thank you for providing additional relevant literature. We have added a sentence regarding the integration of successful analog teaching strategies and digital systems as well as the references to the discussion section.
